# Book Notices

**Published:** 1889-05

**Authors:** 


### BOOK NOTICES.

Lectures on Nervous Diseases from the standpoint of cerebral and spinal localization, and the later methods employed in the diagnosis and treatment of these affections, by Ambrose L. Ranney. A. M., M. D., Professor of the Anatomy and Physiology of the nervous system in the New York Post Graduate Medical School and Hospital; Professor of Nervous and Mental Diseases in the Medical Department of the University of Vermont; Late Adjunct-Professor of Anatomy in the Medical Department of the University of New York; Member of the Neurological Society of New York; Resident Fellow of the New York Academy of Medicine; Member of the New York County Medical Society; Author of the Applied Anatomy of the Nervous System, Electricity in Medicine etc. Profusely illustrated with original diagrams and sketches in color by the Author; carefully selected wood-cuts and reproduced photographs of typical cases, 778 large o. c. pages—price $5.50. F. A. Davis Publisher, No. 1231, Filbert St., Philadelphia, Pa.

The subjects treated in this able and interesting work are as follows:

Sec. 1. Anatomical, Physiological and Pathological deductions respecting the Nerve centres of man.

Sec. 2. The Clinical examination of patients afflicted with nervous diseases, and the steps employed as aids in diagnosis.

Sec. 3. Diseases of the Brain and its envelopes.

Sec. 4. Diseases of the Spinal cord and its envelopes.

Sec. 5. Functional Nervous Diseases.

Sec. 6. Toxic and unclassified nervous diseases.

Sec. 7. Electricity in Medicine, Glossary in Neurological terms.

Under these several heads the many interesting topics connected with the study of the brain and nervous system as now understood are ably and elaborately treated. We commend the work as among the latest and best in this interesting department of Medical Science.

TRANSACTIONS OF THE MEDICAL ASSOCIATION OF GEORGIA.—Our copy of the Georgia Transactions for 1888, containing proceedings and papers of the meeting held in Rome Georgia, was by neglect of the Post Master kept in the Post office until quite recently. Hence we have had no opportunity to notice it heretofore. Its publication by the Secretary was somewhat delayed, as he states, by reason of the failure of the former Secretary to turn over promptly the Records and papers of the Rome meeting.

The Transactions are neatly gotten out by the present Secretary, and the book contains a number of papers which are of much practical interest and importance. Some of these we hope to refer to hereafter.

**Exploration of the Chest in Health and Disease** by Stephen Smith Burt, M. D., Professor of clinical medicine and physical diagnosis in the New York. Post Graduate Medical School and Hospital, Physician to the out-door department Bellevue Hospital, New York. D. Appleton & Co., 1889.

The above work by Prof. Burt was gotten out to aid the student in his efforts to learn the significance of physical signs and their mode of development. The subject is treated under the several heads of

Physical method of Diagnosis; Calormetation; Percussion; Auscultation; Diagnosis by physical signs of Diseases of the Lungs; Pneumonia; Pleurisy; Phthisis Pulmonalis; Exploration of the Heart; Heart Murmurs; Diagnosis by Physical signs of the Heart, and of thoracic Aneurism; Aortic Stenosis. It is a work of 200 pages. Contains a number of beautiful illustrations and much valuable information.

MEDICINE OF THE MIDDLE AGES.—Extracts from LeMoyen Age Medical of Dr. Edmund Dupony, translated by T. C. Minor, M. D., reprinted from the Cincinnati Lancet Clinic, Dec. 1888. Cincinnati Lancet Press Print, 1889.

A book of 99 double column pages, containing numerous facts and incidents connected with the medical history of the middle ages, which are both curious and interesting.

They are given under the following leading heads, viz.:

The physicians of the middle ages; The great epidemics of the middle ages; the Dimonomania of the middle ages; medicine in the literature of the middle ages, etc.

TRANSACTIONS OF THE LOUISIANA STATE MEDICAL SOCIETY at its tenth Annual Session held at Monroe, La., April 25th, 1888.

The above Report of the Transactions of the La. State Medical Society has been so long in reaching us that the time for the meeting of the present year will have passed before we go to press. The officers for 88 –89, Dr. I. J. Newton of Bastrop, President; Drs. D. R. Fox, A. Friedrics of New Orleans; W. D. White of Abbeville; J. W. Allen, Shreveport; T. O. Brewer, Monroe Ducote, Cotton Port, V. Pres. P. B. McCutchen, New Orleans, Secretary.

THE THEORY AND PRACTICE OF OBSTETRICS, including Diseases of Pregnancy and Parturition, Obstetrical operations, etc., by Cazeaux, member of the Imperial Academy of Medicine, adjunct professor in the faculty of medicine, Paris, etc. Remodeled and rearranged, with additions and revisions by S. Tar nier, Professor of Obstetrics and Diseases of Women and Children in the faculty of medicine of Paris. The eighth American edition, edited and revised by Robert J. Hess, M. D. physician to the Northern Dispensary, Philadelphia, with an appendix by Paul F. Munde, M. D., Professor of Gynecology at the New York Polyclinic, and at Dartmouth College; Vice President American gynecological society, etc., with chromo-lithographs and other full page plates, and 175 wood engravings. Philadelphia, P. Blakiston, Son & Co., 1889.

We have here the popular work of Cazeaux, enlarged and revised by able and experienced teachers in the obstetric department. The work is full and elaborate; beautifully illustrated and containing 1202 oc. pages. The department of obstetrics is brought down to its latest advances, and whether as a Text book for students or a work of reference for the practitioner it must be classed with the very ablest and best now extant

In the preface, in which the plan and improvements in the work are ably set forth, Professor Tarnier concludes with the following remark: "I hope therefore, that this book which is, so to speak, a new one, will be found to represent all the most important knowledge which we possess pertaining to the obstetric art.

